# Translation and validation of the Dutch Spine Oncology Study Group Outcomes Questionnaire (SOSGOQ2.0) to evaluate health-related quality of life in patients with symptomatic spinal metastases

**DOI:** 10.1186/s12891-022-05837-1

**Published:** 2022-11-23

**Authors:** Roxanne Gal, Joanne M van der Velden, Daimy C Bach, Jorrit-Jan Verlaan, Ruth E Geuze, Joost PHJ Rutges, Helena M Verkooijen, Anne L Versteeg

**Affiliations:** 1 Division of Imaging and Oncology, University Medical Center Utrecht, University of Utrecht, PO Box 85500, Internal no. E.01.132 3508 GA Utrecht, the Netherlands; 2 Department of Radiation Oncology, University Medical Center Utrecht, Utrecht, the Netherlands; 3 Department of Orthopaedic Surgery, University Medical Center Utrecht, University of Utrecht, Utrecht, the Netherlands; 4 Department of Orthopaedics, Elisabeth Twee Steden Hospital, Tilburg, The Netherlands; 5 Department of Orthopedic Surgery, Erasmus MC, Rotterdam, The Netherlands

**Keywords:** SOSGOQ, Spinal metastases, Patient-reported outcomes, Quality of life, Translation, Validation

## Abstract

**Background:**

The primary goal of palliative treatment of spinal metastases is to maintain or improve health-related quality of life (HRQOL). We translated and validated a Dutch version of The Spine Oncology Study Group Outcome Questionnaire (SOSGOQ2.0), a valid and reliable 20-item questionnaire to evaluate HRQOL in patients with spinal metastases.

**Methods:**

After cross-cultural translation and adaptation, the questionnaire was pre-tested in fifteen patients referred for spine surgery and/or radiotherapy. This resulted in a final questionnaire that was sent to patients for assessment of internal consistency, construct (i.e., convergent and divergent) validity, discriminative power and test-retest reliability.

**Results:**

Overall, 147 patients (mean age 65.6 years, SD = 10.4) completed the questionnaire after a median time of 45.4 months (IQR = 18.9–72.9) after spine surgery and/or radiotherapy. Internal consistency was good for the Physical function, Pain, and Mental health domains (α = 0.87, 0.86, 0.72), but not for Social function (α = 0.04). Good convergent validity was demonstrated except for Social function (r_s _= 0.37 95%CI = 0.21–0.51). Discriminative power between patients with ECOG performance scores of 0–1 and 2–4 was found on all domains and Neurological function items. Test-retest reliability was acceptable for Physical function, Pain and Mental health (ICC = 0.89 95%CI = 0.81–0.94, ICC = 0.88 95%CI = 0.78–0.93, ICC = 0.68 95%CI = 0.48–0.81), whereas ICC = 0.45 (95%CI = 0.17–0.66) for Social function was below threshold. After removing item 20 from the Social function domain, internal consistency improved, and convergent validity and test-retest reliability were good.

**Conclusion:**

The Dutch version of the SOSGOQ2.0 questionnaire is a reliable and valid tool to measure HRQOL in patients with spinal metastases. Item 20 was removed to retain psychometric properties.

**Supplementary Information:**

The online version contains supplementary material available at 10.1186/s12891-022-05837-1.

## Background

The incidence of symptomatic spinal metastases is increasing in patients with advanced cancer. Goals for palliative treatment of spinal metastases include pain relief, local tumor control, maintaining spinal stability, and recovery or preservation of neurological function [1–3]. The overall goal is to preserve or improve independent functioning and health-related quality of life (HRQOL). Evaluation of HRQOL as main treatment outcome is especially important in the setting of palliative treatments such as the treatment of spinal metastases.

Numerous measures to evaluate HRQOL in cancer patients exist, either generic, or tumor- or treatment-specific. The EORTC-QLQ-C15-PAL, EORTC-QLQ-BM22, EQ-5D, FACT-PAL-14 and FACT-BP are commonly used tools to measure quality of life in patients with bone metastases [4–8]. However, these measurement tools are not specific to patients with (symptomatic) spinal metastases as these patients often experience specific complaints such as debilitating back and/or neck pain and neurological deficits (e.g. weakness, sensory loss and difficulty with walking) affecting quality of life [9]. Therefore, generic HRQOL tools for patients with bone metastases may be less sensitive to detect changes in the health status of patients with spinal metastases.

The Spine Oncology Study Group previously developed the Spine Oncology Study Group Outcome Questionnaire (SOSGOQ) to evaluate HRQOL in patients with spinal metastases [10]. Psychometric evaluation resulted in a slightly revised version (SOSGOQ2.0) that is a proven clinically valid and reliable questionnaire [11]. The aim of this study was to translate and validate a Dutch version of the SOSGOQ2.0 questionnaire.

## Methods

### Translation

The original English version of the SOSGOQ2.0 was translated and cross-culturally adapted into Dutch according to the procedure of Beaton et al [12]. First, forward translation from English to Dutch was performed by two native Dutch-speaking researchers (ALV: orthopedic surgery resident and epidemiologist; RG: psychologist and epidemiologist). After synthesis of the forward translations, back translation into English was performed by two native English-speakers. Lastly, an expert committee composed of three translators, a linguist, a methodologist (HMV) and radiation oncology resident (JMV) compared the translations and developed a prefinal version of the questionnaire.

The prefinal questionnaire was pre-tested in fifteen patients referred to University Medical Center (UMC) Utrecht for surgery and/or radiotherapy for (symptomatic) spinal metastases. Patients were asked to say aloud their thoughts while answering the questions of the questionnaire. Follow-up questions were used to obtain additional information about the interpretation and understanding of questions and corresponding answer options. When patients experienced problems in understanding the questionnaire (e.g. difficulties with a specific term or the interpretation of a question), improvements to the questionnaire were made. Improvements that could have resulted in cross-cultural invariance between the original English and translated Dutch version (i.e., the original and translated questionnaire measured not the same construct) were not made but discussed in the Discussion section.

### Patients

For the validation of the SOSGOQ2.0, patients with spinal metastases were recruited from the Departments of Orthopaedic Surgery and Radiation Oncology of the UMC Utrecht, Utrecht, and from the Departments of Orthopaedics of the Erasmus Medical Center, Rotterdam, and Elisabeth-TweeSteden Ziekenhuis (ETZ), Tilburg. Patients in the UMC Utrecht who participated in the prospective PRospective Evaluation of interventional StudiEs on boNe metastases (PRESENT) cohort that includes patients with bone metastases referred to the Department of Radiation Oncology of the UMC Utrecht and who provided consent for collection of patient-reported data, were send questionnaires as described below [13]. Patients in the UMC Utrecht participating in the Metastatic Tumor Research and Outcomes Network (MTRON) that includes patients diagnosed with spinal metastases were approached by a research nurse and asked for consent to participate in the validation study [14]. After providing consent, patients were sent the questionnaire. Patients in the Erasmus Medical Center and ETZ were invited and asked for informed consent by their physician shortly after treatment or during follow-up visits.

### Data collection

Demographic data were extracted from medical records. Together with the SOSGOQ2.0 questionnaire, patients received the 36-Item Short Form Survey (SF-36), the Pain Numeric Rating Scale (NRS) measuring average pain on a scale from 0 (no pain) to 10 (worst pain), a global QOL rating scale measuring self-reported health status ranging from 0 to 10, and the Eastern Cooperative Oncology Group (ECOG) performance score to assess disease progression. Questionnaires were completed on paper or online, depending on the preference of the patient.

The SOSGOQ2.0 questionnaire is a 20-item questionnaire containing four domains (Physical function, Pain, Mental health, Social function) and four single items (Neurological function of legs, arms, bladder and bowel) [11]. In addition, seven post-therapy items should be used during follow-up in combination with the first 20 items. Each item has five response options. The sum of the items within a domain and the single items were transformed to a 0-100 scale. A higher score on the Physical and Social function domains indicated a higher level of functioning, and a higher score on the Pain and Mental health domains and the Neurological function items indicated a lower level of functioning or neurological symptoms.

The SF-36 is a self-administered 36-item questionnaire with standardized response choices that measures functional status and well-being on eight domains: Physical function, Bodily pain, Role limitations due to physical problems, Role limitations due to emotional problems, Mental health, Social functioning, Vitality, and General health perception [15]. In addition, a single item measured perceived change in health. Transformed domain scores ranged on a scale between 0 and 100, where a higher score represented a better health status.

With the ECOG performance score, patients could indicate their level of functioning in terms of ability to care for themselves, daily activity and physical ability, ranging from 0 (Fully active, able to carry on all pre-disease performance without restriction) to 4 (Completely disabled, cannot carry on any self-care, totally confined to bed or chair) [16].

Patients could indicate whether they were willing to complete a retest questionnaire. Upon agreement, patients received the SOSGOQ2.0 questionnaire.

### Statistical analyses

A minimum sample size of 140 was considered adequate for validation of this 20-item questionnaire (excluding the post-therapy questions) according to Terwee et al., who required a sample size with a 1:7 patient-to-item ratio or ≥ 100 patients to evaluate measurement properties of health status questionnaires [17].

Statistical analyses were performed using R statistical software version 1.3.1093. Demographic data were summarized using descriptive statistics. Measurement properties of post-therapy items were not evaluated.

#### Internal consistency

Internal consistency, the extent to which items are interrelated within a domain, was assessed using Cronbach’s alpha coefficient. A Cronbach’s alpha between 0.70 and 0.95 was considered reflecting good internal consistency [17]. Correlations < 0.70 indicated inconsistency and values > 0.90 indicated redundancy of questionnaire items. Also, Spearman’s rank correlation coefficients were used to assess correlations between SOSGOQ2.0 items and its own domain score.

#### Construct validity: convergent and divergent validity

Convergent validity, the extent to which the SOSGOQ2.0 domains are related to other measures that measure the same construct, was assessed using Spearman’s rank correlation coefficients between SOSGOQ2.0, the SF-36 domains and the Pain NRS that conceptually measure the same construct. It was hypothesized that large correlations will be found of the SOSGOQ2.0 Physical function with the SF-36 Physical functioning and Role-Physical domains, of the SOSGOQ2.0 Pain domain with the SF-36 Bodily pain domain and Pain NRS, of the SOSGOQ2.0 Mental health domain with the SF-36 Role emotional and Mental health domains, and of the SOSGOQ2.0 Social function domain with the SF-36 Social functioning domain. Divergent validity, the extent to which the SOSGOQ2.0 domains are not related to measures other constructs, was assessed using Spearman’s rank correlation coefficients between SOSGOQ2.0 domains and domains that conceptually measure different constructs. It was hypothesized that small or medium correlations would be found between domains that were not stated above. Spearman’s rank correlation coefficients less than 0.30 were considered small, between 0.30 and 0.49 medium, and 0.50 or greater large [18].

#### Discriminative power

The ability to discriminate between patients with an ECOG performance score of 0–1 and 2–4 was evaluated by comparing the domain scores and four Neurological function items scores with the independent samples t-test. Floor and ceiling effects, i.e., scores cluster at the worst or best score resulting in little variance, were considered to be present when 15% of the patients had the lowest (0) or highest (100) score on one of the SOSGOQ2.0 domains.

#### Test-retest reliability

Test-retest reliability of the SOSGOQ2.0 domains and four Neurological function items was evaluated using the Intraclass Correlation Coefficient (ICC) between the initial and retest questionnaire. A two-way mixed effect model was used to calculate the ICC. An ICC ≥ 0.70 indicates acceptable or good reliability [17]. Only those patients who completed the retest questionnaire within two weeks after completing the baseline questionnaire were included.

## Results

### Translation

No problems were encountered during translation. Pre-tests of the pre-final version of the questionnaire did not result in amendments to the text.

### Study population

Of the 219 patients who consented for collection of patient-reported data (PRESENT: *n* = 166) or participation in this study (i.e., MTRON, EMC and ETZ: *n* = 53), 147 patients (67%) completed the questionnaire (PRESENT: *n* = 101/166 [61%]; MTRON/EMC/ETZ: *n* = 44/51 [86%]). Of these patients, 43 (29%) completed the retest questionnaire within two weeks. Mean age was 65.6 years (SD = 10.4, range = 32–92) and 48% (*n* = 71) were female (Table 1). The most common primary tumor was breast (*n* = 49, 33%), followed by prostate (*n* = 36, 25%) and lung (*n* = 25, 17%). Median time since diagnosis of the primary tumor was 26.2 months (IQR = 15.7–67.9) and median time since spine surgery and/or radiotherapy was 10.5 months (IQR = 4.3–16.8), whereby 31% of the patients were treated in the last 6 months and 22% 6–12 months ago.

**Table 1:** Baseline characteristics of patients who completed the Dutch SOSGOG2.0 questionnaire

No. patients	147
Age in years, mean (SD)	65.6 (10.4)
Sex, female, n (%)	71 (48.3)
Primary tumor, n (%)	
Breast	49 (33.3)
Prostate	36 (24.5)
Lung	25 (17.0)
Multiple myeloma	17 (11.6)
Other	20 (13.6)
Time since diagnosis of primary tumor in months, median (IQR)^a^	26.2 (15.7–67.9)
Location of spinal metastasis, n (%)^b,c^	
Cervical	23 (15.6)
Thoracic	80 (54.4)
Lumbar	70 (47.6)
Sacral	19 (12.9)
Treatment of spinal metastasis, n (%)	
No treatment	2 (1.4)
Radiotherapy only	85 (57.8)
Surgery only	15 (10.2)
Radiotherapy and surgery	45 (30.6)
Time since start of treatment in months, median (IQR)	10.5 (4.3–16.8)

*Abbbreviations*: *IQR* Interquartile range, *No* Number, *SD* Standard deviation

^a^Information not available from 24 (16.3%) patients

^b^Information not available from 6 (4.1%) patients, or patient received no treatment

^c^Percentages do not add up to 100% as patients could have more than one location

### Internal consistency

The Cronbach’s alpha for the Physical function, Pain and Mental health domains was 0.87, 0.86 and 0.72, respectively, indicating good internal consistency (Table 2). In contrast, the Social function domain showed low internal consistency (α = 0.04). The correlation between item 20 (“Do you feel comfortable meeting new people?”) and the Social function domain score was small (r_s _= 0.16). After removing item 20, the Social function showed greater internal consistency yet still below the threshold (α = 0.68).

**Table 2:** Internal consistency and item-domain correlations of the Dutch SOSGOQ2.0 domains

			**Physical function**	**Pain**	**Mental health**	**Social function**	**Social function (without item 20)**
**Internal consistency**, α^a^			0.87	0.86	0.72	0.04	0.68
**Item-domain correlations,** r_s_	**N**	**Item – Own domain**	**Item – Other domains**				
**Physical function**							
1 Level of activity	147	0.80^*^	-	0.44^*^	0.25^*^	0.29^*^	0.38^*^
2 Ability to work	145	0.85^*^	-	0.44^*^	0.38^*^	0.25^*^	0.39^*^
3 Limited ability to care for yourself	147	0.70^*^	-	0.50^*^	0.26^*^	0.39^*^	0.49^*^
4 Assistance to travel outside	147	0.82^*^	-	0.44^*^	0.26^*^	0.37^*^	0.44^*^
5 Assistance walking	146	0.80^*^	-	0.43^*^	0.23^**^	0.33^*^	0.42^*^
6 Social functions	147	0.69^*^	-	0.44^*^	0.25^*^	0.22^**^	0.39^*^
**Pain**							
11 Back/neck pain	147	0.83^*^	0.43^*^	-	0.42^*^	0.47^*^	0.59^*^
12 Pain in most comfortable position	147	0.82^*^	0.42^*^	-	0.40^*^	0.53^*^	0.65^*^
13 Limiting mobility	147	0.84^*^	0.53^*^	-	0.49^*^	0.45^*^	0.62^*^
14 Ability to manage pain	146	0.80^*^	0.49^*^	-	0.43^*^	0.31^*^	0.47^*^
15 Overwhelming pain	147	0.79^*^	0.45^*^	-	0.38^*^	0.32^*^	0.44^*^
**Mental health**							
16 Depression	147	0.87^*^	0.31^*^	0.39^*^	-	0.19^**^	0.40^*^
17 Anxiety	146	0.89^*^	0.33^*^	0.52^*^	-	0.32^*^	0.49^*^
**Social function**							
18 Concentration	146	0.74^*^	0.50^*^	0.66^*^	0.50^*^	^−^	0.87^*b^
19 Personal relationships	145	0.76^*^	0.41^*^	0.52^*^	0.38^*^	-	0.87^*b^
20 Meeting new people	145	0.16	-0.32^*^	-0.37^*^	-0.46^*^	-	-
**Neurological function items**							
7 Neurol. function – legs	147	-	0.44^*^	0.35^*^	0.15	0.20^**^	0.20^**^
8 Neurol. function – arms	147	-	0.39^*^	0.38^*^	0.20^**^	0.29^*^	0.29^*^
9 Neurol. function – bowel	147	-	0.16	0.11	0.02	-0.02	0.00
10 Neurol. function – bladder	146	-	0.13	0.04	0.09	0.05	0.07

*Abbbreviations*: *Neurol.* Neurological

^a^α 0.70–0.95 indicates good internal consistency

^b^Item – Own domain correlation

^*^ *p* < 0.001; ^**^ *p* < 0.05

### Construct validity: convergent and divergent validity

All correlations that were hypothesized to measure the same construct were above 0.50, except for the SOSGOQ2.0 Social function domain with the SF-36 Social functioning domain, which was 0.37 (95%CI = 0.21–0.51; Table 3). After removing item 20, this correlation improved to a large correlation of 0.54 (95%CI = 0.39–0.67).

Although not hypothesized, strong but lower correlations were found between the SOSGOQ2.0 Physical function domain and all domains of the SF-36 and Pain NRS, between the SOSGOQ2.0 Pain domain and the SF-36 Physical functioning, Role Physical, Vitality, Social functioning, Role Emotional and Mental health domains, and between the SOSGOQ2.0 Mental health domain and the SF-36 Vitality domain. Weak and medium correlations were found between the other SOSGOQ2.0 domains and SF-36 domains and Pain NRS. However, the SOSGOQ2.0 Social function domain showed strong correlations with the SF-36 Role Physical, Bodily pain and Vitality domains and Pain NRS after removing item 20.

**Table 3:** Convergent and divergent validity a of the SOSGOQ2.0 domains and Neurological function items^a^

	**Physical function**	**Pain**	**Mental health**	**Social function**	**Neurol. function: legs**	**Neurol. function: arms**	**Neurol. function: bowel**	**Neurol. function: bladder**	**Social function (without item 20)**
**SF-36**									
Physical functioning	0.80 (0.71–0.86)^b^	0.55 (0.40–0.67)	0.36 (0.18–0.51)	0.30 (0.14–0.44)	0.61 (0.49–0.71)	0.38 (0.22–0.53)	0.20 (0.02–0.36)	0.14 (-0.02–0.30)	0.43 (0.27–0.57)
Role Physical	0.78 (0.69–0.86)^b^	0.60 (0.46–0.71)	0.41 (0.25–0.55)	0.44 (0.30–0.57)	0.44 (0.29–0.58)	0.41 (0.27–0.54)	0.12 (-0.05–0.27)	0.10 (-0.06–0.27)	0.57 (0.43–0.68)
Bodily pain	0.67 (0.55–0.77)	0.82 (0.75–0.87)^b^	0.42 (0.25–0.56)	0.47 (0.34–0.58)	0.35 (0.20–0.49)	0.45 (0.30–0.58)	0.12 (-0.05–0.27)	0.04 (-0.12–0.21)	0.62 (0.50–0.72)
General health	0.48(0.34–0.60)	0.37 (0.19–0.51)	0.41 (0.26–0.55)	0.19 (0.03–0.34)	0.25 (0.08–0.40)	0.34 (0.20–0.48)	-0.03 (-0.18–0.12)	0.06 (-0.09–0.22)	0.34 (0.16–0.49)
Vitality	0.64 (0.51–0.74)	0.61 (0.49–0.71)	0.56 (0.43–0.67)	0.31 (0.17–0.44)	0.31 (0.15–0.45)	0.42 (0.28–0.56)	0.03 (-0.13–0.19)	0.13 (-0.03–0.29)	0.50 (0.36–0.61)
Social functioning	0.59 (0.44–0.71)	0.53 (0.39–0.64)	0.46 (0.32–0.61)	0.37 (0.21–0.51)^b^	0.25 (0.09–0.41)	0.32 (0.16–0.47)	0.11 (-0.05–0.26)	0.15 (-0.01–0.34)	0.54 (0.39–0.67)^b^
Role Emotional	0.48 (0.32–0.62)	0.45 (0.28–0.58)	0.53 (0.39–0.65)^b^	0.19 (0.02–0.35)	0.26 (0.10–0.43)	0.19 (0.03–0.34)	0.17 (0.00–0.33)	0.17 (0.00–0.34)	0.41 (0.26–0.55)
Mental Health	0.50 (0.36–0.63)	0.52 (0.38–0.65)	0.71 (0.59–0.79)^b^	0.23 (0.07–0.37)	0.19 (0.02–0.35)	0.25 (0.09–0.39)	0.11 (-0.08–0.27)	0.11 (-0.05–0.27)	0.46 (0.31–0.58)
**Pain NRS**									
Average daily pain	-0.56 (-0.68–0.42)	-0.86 (-0.90–0.80)^b^	-0.38 (-0.52–0.22)	-0.38 (-0.51–0.23)	-0.38 (-0.51–0.22)	-0.38 (-0.51–0.24)	-0.17 (-0.32–0.02)	-0.15 (-0.32–0.02)	-0.57 (-0.67–0.44)

*Abbbreviations*: *Neurol.* Neurological, *NRS* Numeric Rating Scale, *SF-36* 36-item Short Form Survey

^a^Spearman’s rank correlation coefficient (95%CI); r_s_ <0.30 = small, r_s_ 0.30-0.49 = medium, r_s_ >0.50 = large

^b^Hypothesized that domains measure the same construct

### Discriminative power

The independent samples t-test showed significantly higher scores for the patients with an ECOG performance score of 0–1 on all SOSGOQ2.0 domains and the Neurological function of legs and arms items compared to patients with an ECOG performance score of 2–4 (Table 4). For example, patients with an ECOG performance score 0–1 had a mean score of 79.9 (SD = 14.6) and 72.6 (SD = 19.0) on the Physical function and Pain domains compared to a mean score of 49.5 (SD = 17.0) and 50.0 (SD = 21.2) in patients with an ECOG performance score of 2–4. No statistically significant difference was found on the items Neurological function of the bowel and bladder. No floor or ceiling effects were found. However, a ceiling effect was observed in the Social function domain after removing item 20; 43 patients (29.3%) achieved the highest possible score.

**Table 4:** Means and standard deviations, discriminative power and test–retest reliability of the Dutch SOSGOQ2.0

	**All patients**					**ECOG 0–1**			**ECOG 2–4**					
	**N**	**Mean**	**SD**	**Floor, n (%)**	**Ceiling, n (%)**	**N**	**Mean**	**SD**	**N**	**Mean**	**SD**	**t-test**^**a**^	***p***	**Test–retest reliability, ICC (95%CI)**^**b,c**^
Physical function	147	67.0	22.0	0 (0.0)	5 (3.4)	86	79.9	14.6	59	49.5	17.0	11.9	< 0.001	0.89 (0.81–0.94)
Pain	147	63.5	22.8	0 (0.0)	7 (4.8)	86	72.6	19.0	59	50.0	21.2	6.7	< 0.001	0.88 (0.78–0.93)
Mental health	147	68.6	21.4	0 (0.0)	21 (14.3)	86	74.1	19.3	59	59.8	21.5	4.2	< 0.001	0.68 (0.48–0.81)
Social function	145	58.9	14.9	0 (0.0)	2 (1.4)	85	62.8	12.1	58	52.9	16.8	4.1	< 0.001	0.45 (0.17–0.66)
Neurol. Legs	147	3.7	1.2	-	-	86	4.1	1.0	59	3.3	1.4	4.1	< 0.001	0.82 (0.69–0.90)
Neurol. Arms	147	4.3	0.9	-	-	86	4.6	0.7	59	3.9	1.1	4.9	< 0.001	0.68 (0.48–0.81)
Neurol. Bowel	147	4.7	0.6	-	-	86	4.7	0.6	59	4.7	0.6	0.5	0.63	0.51 (0.25–0.70)
Neurol. Bladder	146	4.2	1.0	-	-	86	4.2	1.0	58	4.2	1.0	-0.1	0.96	0.84 (0.72–0.91)
Social function (without item 20)	144	75.0	22.8	1 (0.7)	43 (29.3)	85	83.2	17.8	57	62.1	23.7	6.1	< 0.001	0.73 (0.55–0.84)

*Abbbreviations*: *ECOG* Eastern Cooperative Oncology Group, *ICC* Intraclass correlation coefficient, *Neurol.* Neurological, *SD* Standard deviation

^a^Independent sample t-test result comparing patients with an ECOG performance score of 0–1 and 2–4 on SOSGOQ2.0 domains and Neurological function items

^b^*n* = 43

^c^ICC ≥ 0.70 = acceptable or good reliability

### Test-retest reliability

In total, 43 (29.3%) patients completed the retest questionnaire after a median of 9 days (range = 5–14). The Physical function and Pain domains showed an acceptable test-retest reliability (ICC = 0.89 95%CI = 0.81–0.94 and ICC = 0.88 95%CI = 0.78–0.93 respectively; Table 4). An ICC of 0.68 (95%CI = 0.48–0.81) was found for the Mental health domain. An ICC below the threshold (ICC = 0.45 95%CI = 0.17–0.66) was found for the Social function domain, which increased to an acceptable test-retest reliability after removing item 20 (i.e. “Are you comfortable meeting new people?”; ICC = 0.73 95%CI = 0.55–0.84).

## Discussion

In this study, the Spine Oncology Study Group Outcomes Questionnaire 2.0 (SOSGOQ2.0) was translated from English and cross-culturally adapted into Dutch, and psychometric properties of the translated questionnaire were evaluated. After removing item 20, the results showed that the reliability and validity of the SOSGOQ2.0 were acceptable and hence, that the SOSGOQ2.0 is a reliable and valid tool to measure HRQOL in Dutch patients with spinal metastases.

The method of Beaton was used to translate and cross-culturally adapt the questionnaire to provide a HRQOL questionnaire that is culturally appropriate for Dutch patients with spinal metastases [12]. At the same time, it was important to preserve equivalence between the original English version and the translated Dutch version, ensuring that psychometric properties were maintained and that both questionnaires measure the same construct.

Internal consistency and test-retest reliability of the Social function domain were low, as well as convergent validity (i.e., medium correlation between the SOSGOQ2.0 Social function and SF-36 Social functioning domains). This may be explained by item 20 (“Are you comfortable meeting new people?” which was translated to “Voelt u zich op uw gemak als u nieuwe mensen ontmoet?”), that correlated low with the SOSGOQ2.0 Social function domain score and negatively with the other SOSGOQ2.0 domain scores. Differences in psychometric properties between the original and translated questionnaire may be ascribed to cultural differences. In the pre-test phase (interviews), most patients indicated that their medical condition did not interfere in feeling comfortable meeting new people. Also, inappropriate translation of the question may impair psychometric properties. However, cross-cultural translation was carried out carefully according to Beaton et al. with multiple translators, and extensively discussed and approved by a panel of experts [12].

To allow comparison of results across different translated versions, we did not remove this item from the questionnaire after the pre-test phase. However, based on the psychometric properties and input from the pre-test phase, analyses were repeated after removing item 20 from the questionnaire. After removing item 20, internal consistency improved yet not above the threshold, and test-retest reliability was acceptable. Convergent validity of the SOSGOQ2.0 Social function domain improved from medium to large correlation with the Social functioning domain of the SF-36, indicating that the SOSGOQ2.0 is a better approximation of the construct social function after removing item 20. As psychometric properties improved after removing item 20, we decided to remove the item from the questionnaire. Although the structure of the translated questionnaire differs from the original questionnaire, the original questionnaire does not require revisions as psychometric properties of the original questionnaire demonstrated to be good [11, 19].

Strong correlations were found between the SOSGOQ2.0 Physical function and Pain domains and the SF-36 Physical functioning, Role Physical and Vitality domains. Strong but lower correlations were found with the SF-36 Social functioning, Role Emotional and Mental health domains. Although not hypothesized that these scales measured the same construct, it is likely that these scales correlate well as painful spinal metastases significantly impact physical and daily functioning, and mental health [20].

The four domains and the Neurological function of legs and arms items were able to discriminate between patients with an ECOG performance score of 0–1 and 2–4. This score assesses a patient’s level of functioning and ability to care for themselves, which is an important predictor of HRQOL [21, 22]. This implies that the Dutch SOSGOQ2.0 questionnaire is able to detect differences or changes in symptoms that affect HRQOL, which is important in prospective studies examining the impact of an intervention. In addition, HRQOL is one of the most important outcomes in the setting of palliative treatment. Therefore, application of the SOSGOQ2.0 has the potential to support routine clinical practice, including facilitation of physician-patient counseling, treatment choices, and monitoring of changes after treatment. However, future research is needed to determine the minimal clinically important difference (MCID), the minimal change considered relevant by patients and physicians.

In the original SOSGOQ2.0 questionnaire, it was not specified whether patients should take into account the effect of opioid pain medication while completing the questionnaire. During the pre-test phase, it turned out that some patients had trouble answering some questions as pain interference with daily functioning was dependent on the opioid pain medications they took. However, it was assumed that patients consistently answered all questions while taking into account the effect of opioid pain medication, while others consistently answered all questions while not taking into account this effect. Therefore, this should not have affected evaluation of the validity of the questionnaire. Use of opioid pain medication should be taken into account when utilizing and interpreting the questionnaire in clinical practice. In addition, when developing a new questionnaire, it should be specified whether patients should take into account the effect of opioid pain medication or not. This is generally not specified in questionnaires but especially important in patients with painful bone metastases.

Two other issues were observed in the pre-test phase, but no corrections were made as this would hamper cross-cultural equivalence between the original and translated questionnaire. First, patients who were retired had no opportunity to indicate this when answering Item 2 (“What is your ability to work (including at home/study)?”). Instead, they gave an indication of their ability, but this might be inaccurate. Second, items 11 and 19 (“Overall, on average, how much back/neck pain do you have?” and “Do you feel that your spine condition affects your personal relationships?”) were double barreled and answers differed for their back and neck, or for romantic relationships and family/peer relationships.

Strengths of this study were following standard guidelines for the translation of the questionnaire, and inclusion of a heterogeneous group of patients that differed in time since diagnosis. However, we acknowledge the limitations that need to be taken into account. This study was conducted during the Covid-19 outbreak in 2020. Restrictions aiming to limit the spread of the virus may have impacted the results, e.g. social distancing, travel restrictions, maximum number of daily visitors at home, and closing public places. This may have had impact on answers to particular questions, for example some patients may have answered “Never” or “Rarely” on item 6 (“Do you leave the house for social functions?”) due to Covid restrictions, but should have answered “Often” or “Very often” without Covid restrictions because they were not limited by their medical condition. This may also have had impact on item 20. However, the pre-test phase showed that answers of most patients were not influenced by these restrictions. A second limitation might be the response rate of 61% (*n* = 101/166) among patients who participated in the PRESENT cohort. In addition, 86% of the participants (*n* = 44/51) who first consented for participation in this study completed the questionnaire. Patients who provided consent and completed the questionnaire might differ from non-participants and non-responders, e.g. younger patients with a better medical condition might be more likely to complete the questionnaire. Therefore, information about means and standard deviations should be interpreted with care.

## Conclusion

The Dutch version of the SOSGOQ2.0 questionnaire is a reliable and valid questionnaire to measure HRQOL in patients with spinal metastases. This questionnaire can be used in the clinical setting for facilitation of physician-patient counseling, treatment choices, and monitoring of changes after treatment, and in the research setting examining the impact of an intervention.

## Supplementary Information

**Additional file 1.** Final version of the Dutch Spine Oncology Study Group Outcomes Questionnaire 2.0 (SOSGOQ2.0).

**Additional file 2.** Scoring manual of the Dutch Spine Oncology Study Group Outcomes Questionnaire 2.0 (SOSGOQ2.0).

## Data Availability

The dataset generated and/or analysed during the current study is available from the corresponding author on reasonable request.
