# Supplementary material for: Translation and validation of the Dutch Spine Oncology Study Group Outcomes Questionnaire (SOSGOQ2.0) to evaluate health-related quality of life in patients with symptomatic spinal metastases
Source: BMC Musculoskelet Disord. 2022 Nov 23;23:1009. doi: 10.1186/s12891-022-05837-1 (PMC9686456; doi:10.1186/s12891-022-05837-1)
Supplement: Supplementary file 1 — Additional file 1. Final version of the Dutch Spine Oncology Study Group Outcomes Questionnaire 2.0 (SOSGOQ2.0). [file 12891_2022_5837_MOESM1_ESM.pdf]

Spine Oncology Study Group Outcomes Questionnaire 2.0
(SOSGOQ2.0)

Instructie: Denk bij het invullen van deze vragenlijst aan uw huidig functioneren en de klachten in de afgelopen 4 weken. Kies **ÉÉN ANTWOORD** bij elke vraag. Vraag 20-26 hoeven alleen ingevuld te worden **NA** de behandeling voor de tumor in uw wervelkolom.

Naam patiënt: ______________________________

Datum (DD/MM/JJ): _____ / _____ / _____

Patiënt nummer: ____________________________
(wordt ingevuld door de arts)

In te vullen door de patiënt

1. Hoe actief bent u in het dagelijks leven?
- ☐ Volledig actief zonder beperkingen
- ☐ Beperkte activiteiten buitenshuis
- ☐ Activiteiten beperkt tot binnenshuis
- ☐ Beperkt tot liggende en zittende activiteiten
- ☐ Ik ben aan bed gebonden

2. Hoe lang kunt u werken of studeren op een dag? (inclusief werken vanuit huis)
- ☐ Ik ervaar geen beperkingen
- ☐ Meer dan 4 uur, maar minder dan 8 uur
- ☐ Meer dan 2 uur, maar minder dan 4 uur
- ☐ Minder dan 2 uur
- ☐ Ik kan niet werken of studeren

3. Beperkt uw wervelkolom u om voor uzelf te zorgen?
- ☐ Helemaal niet
- ☐ Een beetje
- ☐ Matig
- ☐ Veel
- ☐ Heel veel

4. Heeft u hulp van anderen nodig om naar buiten te gaan?
- ☐ Nooit
- ☐ Bijna nooit
- ☐ Soms
- ☐ Vaak
- ☐ Heel vaak

5. Welke hulp heeft u nodig bij het lopen?
- ☐ Geen
- ☐ Een wandelstok/kruk
- ☐ Een rollator/twee krukken
- ☐ Hulp van anderen
- ☐ Ik kan niet lopen

6. Verlaat u het huis voor sociale activiteiten? (bijvoorbeeld familie/vrienden bezoeken, uitstapjes naar het theater of de bioscoop, naar een feestje)
- ☐ Nooit
- ☐ Bijna nooit
- ☐ Soms
- ☐ Vaak
- ☐ Heel vaak

7. Heeft u krachtsverlies in uw benen?
- ☐ Helemaal niet
- ☐ Soms, een beetje
- ☐ Altijd, een beetje
- ☐ Altijd, matig
- ☐ Altijd, ernstig

8. Heeft u krachtsverlies in uw armen?
- ☐ Helemaal niet
- ☐ Soms, een beetje
- ☐ Altijd, een beetje
- ☐ Altijd, matig
- ☐ Altijd, ernstig

9. Verliest u wel eens ongewild ontlasting?
- ☐ Nooit
- ☐ Bijna nooit
- ☐ Soms
- ☐ Vaak
- ☐ Heel vaak

10. Heeft u problemen met de controle over uw blaas?
- ☐ Nooit
- ☐ Bijna nooit
- ☐ Soms
- ☐ Vaak
- ☐ Ik moet gekatheteriseerd worden

11. Hoeveel pijn heeft u meestal in uw rug/nek?
- ☐ Geen
- ☐ Erg weinig
- ☐ Weinig
- ☐ Matig
- ☐ Veel

12. Als u in uw meest prettige houding zit/ligt, heeft u dan nog steeds pijn in uw rug/nek (waardoor u niet kunt slapen)?
- ☐ Nooit
- ☐ Bijna nooit
- ☐ Soms
- ☐ Vaak
- ☐ Heel vaak

13. Hoe vaak heeft u door uw pijn problemen met zitten, staan en/of lopen?

- ☐ Nooit
- ☐ Bijna nooit
- ☐ Soms
- ☐ Vaak
- ☐ Heel vaak

14. Hoeveel vertrouwen heeft u erin dat u zelf uw pijn onder controle kunt krijgen?

- ☐ Geen vertrouwen
- ☐ Weinig vertrouwen
- ☐ Matig vertrouwen
- ☐ Veel vertrouwen
- ☐ Heel veel vertrouwen

15. Als ik pijn heb, dan is het vreselijk en kan ik nergens anders aan denken.

- ☐ Nooit
- ☐ Bijna nooit
- ☐ Soms
- ☐ Vaak
- ☐ Heel vaak

16. Bent u wel eens neerslachtig?

- ☐ Nooit
- ☐ Bijna nooit
- ☐ Soms
- ☐ Vaak
- ☐ Heel vaak

17. Roept het probleem van uw wervelkolom gevoelens van angst op?

- ☐ Nooit
- ☐ Bijna nooit
- ☐ Soms
- ☐ Vaak
- ☐ Heel vaak

18. Hebben de problemen in uw wervelkolom invloed op uw concentratie bij gesprekken, lezen en tv kijken?

- ☐ Nooit
- ☐ Bijna nooit
- ☐ Soms
- ☐ Vaak
- ☐ Heel vaak

19. Hebben de problemen in uw wervelkolom invloed op uw persoonlijke relaties? (bijvoorbeeld uw liefdesrelatie, familie, vrienden)

- ☐ Nooit
- ☐ Bijna nooit
- ☐ Soms
- ☐ Vaak
- ☐ Heel vaak

Vul deze vragen alleen in na de behandeling

20. Bent u tevreden over de resultaten van de behandeling van de tumor in uw wervelkolom?

- ☐ Erg tevreden
- ☐ Een beetje tevreden
- ☐ Niet tevreden en niet ontevreden
- ☐ Een beetje ontevreden
- ☐ Erg ontevreden

21. Zou u dezelfde behandeling voor de tumor in uw wervelkolom weer kiezen?

- ☐ Zeker
- ☐ Waarschijnlijk
- ☐ Dat weet ik niet
- ☐ Waarschijnlijk niet
- ☐ Zeker niet

22. Kunt u door de behandeling van de tumor in uw wervelkolom beter bewegen en lukt het u om uw dagelijkse activiteiten beter uit te voeren?

- ☐ Veel beter
- ☐ Iets beter
- ☐ Geen verandering
- ☐ Iets slechter
- ☐ Veel slechter

23. Hoe heeft de behandeling van de tumor in uw wervelkolom de kracht in uw armen/benen en/of de controle over uw blaas/darmen beïnvloed?

- ☐ Veel beter
- ☐ Iets beter
- ☐ Geen verandering
- ☐ Iets slechter
- ☐ Veel slechter

24. Hoe heeft de behandeling de pijnklachten vanuit uw rug beïnvloed?

- ☐ Veel beter
- ☐ Iets beter
- ☐ Geen verandering
- ☐ Iets slechter
- ☐ Veel slechter

25. Hoe heeft de behandeling van uw wervelkolom uw gevoelens van neerslachtigheid en angst veranderd?

- ☐ Sterk verbeterd
- ☐ Een beetje verbeterd
- ☐ Geen verandering
- ☐ Een beetje verslechterd
- ☐ Sterk verslechterd

26. Hoe heeft de behandeling van de tumor in uw wervelkolom uw mogelijkheid veranderd om sociaal actief te zijn? (bijvoorbeeld familie/vrienden bezoeken, uitstapjes naar het theater of de bioscoop, naar een feestje)

- ☐ Veel beter
- ☐ Iets beter
- ☐ Geen verandering
- ☐ Iets slechter
- ☐ Veel slechter
