# Supplementary material for: Translation and validation of the Dutch Spine Oncology Study Group Outcomes Questionnaire (SOSGOQ2.0) to evaluate health-related quality of life in patients with symptomatic spinal metastases
Source: BMC Musculoskelet Disord. 2022 Nov 23;23:1009. doi: 10.1186/s12891-022-05837-1 (PMC9686456; doi:10.1186/s12891-022-05837-1)
Supplement: Supplementary file 2 — Additional file 2. Scoring manual of the Dutch Spine Oncology Study Group Outcomes Questionnaire 2.0 (SOSGOQ2.0). [file 12891_2022_5837_MOESM2_ESM.pdf]

Handleiding berekenen score SOSGOQ2.0 – Nederlandse versie

Domein	Items
Fysiek functioneren	1*, 2*, 3*, 4*, 5*, 6
Pijn	11*, 12*, 13*, 14, 15*
Mentale gezondheid	16*, 17*
Sociaal functioneren	18*, 19*, 20*
**Single items betreffende neurologisch functioneren**	
Benen	7*
Armen	8*
Blaas	9*
Darmfunctie	10*

* Deze items moeten omgescoord worden (‘1’ → ‘5’, ‘2’ → ‘4’, ‘4’ → ‘2’, ‘5’ → ‘1’)

Berekenen van de domein score

De domeinscore wordt omgerekend naar een 0-100 schaal met behulp van de volgende algoritme:

$$\frac{ruwe\ score - laagst\ mogelijke\ ruwe\ score}{hoogst\ mogelijke\ ruwe\ score - laagst\ mogelijke\ ruwe\ score}\ x\ 100$$

Ruwe score = som van de items binnen het domein

Een domein score kan berekend worden als een patiënt minstens vijftig procent van de items binnen een domein (of de helft van de items plus één bij een even aantal items).

Een hogere score correspondeert met een beter niveau van functioneren voor de domeinen fysiek en sociaal functioneren. Een hogere score correspondeert met een mindere mate van symptomen voor de domeinen pijn en mentale gezondheid.

Items betreffende neurologisch functioneren

De items betreffende het neurologisch functioneren zijn single symptoom items (items 7-10). Een score op een 0-100 schaal wordt met behulp van de volgende algoritme berekend:

$$\frac{antwoord - laagst\ mogelijke\ antwoord}{hoogst\ mogelijke\ antwoord - laagst\ mogelijke\ antwoord}\ x\ 100$$

Laagst mogelijke antwoord = 1, hoogst mogelijke antwoord = 5

Een verandering van 25 punten in de score komt overeen met een verandering van 1 punt in de antwoordmogelijkheden.

Een hogere score correspondeert met een slechter niveau van neurologisch functioneren.

Een domein score kan berekend worden om rapportage te vergemakkelijken. Voor de interpretatie van de domein score moeten de items los van elkaar geïnterpreteerd worden om de aard van de neurologische problemen vast te stellen.

Totaalscore voor de SOSGOQ2.0

Een totaalscore voor de SOSGOQ2.0 wordt berekend met behulp van de domeinen fysiek functioneren, pijn, mentale gezondheid en sociaal functioneren:

$$\frac{Fysiek\ functioneren + Pijn + Mentale\ gezondheid + Sociaal\ functioneren}{4}$$

Als één van de domeinscores niet berekend kan worden door missende items, kan de totaalscore ook niet berekend worden.

Items na de behandeling

De items na de behandeling zijn single items (item 20-26). Een score op een 0-100 schaal kan met behulp van de volgende algoritme berekend worden:

$$\frac{antwoord - laagst\ mogelijke\ antwoord}{hoogst\ mogelijke\ antwoord - laagst\ mogelijke\ antwoord}\ x\ 100$$

Laagst mogelijke antwoord = 1, hoogst mogelijke antwoord = 5

Een verandering van 25 punten in de score komt overeen met een verandering van 1 punt in de antwoordmogelijkheden.

Een hogere score correspondeert met een hogere tevredenheid en een verbetering na de behandeling.

Het gebruik van deze items is aanbevolen tijdens de follow-up, in combinatie met de basis items (items 1-19) die verschillende aspecten van de kwaliteit van leven evalueren.
